# "CAN Stop" - Implementation and evaluation of a secondary group prevention for adolescent and young adult cannabis users in various contexts - study protocol

**DOI:** 10.1186/1472-6963-11-80

**Published:** 2011-04-18

**Authors:** Christiane Baldus, Alejandra Miranda, Nina Weymann, Olaf Reis, Kerstin Moré, Rainer Thomasius

**Affiliations:** 1 German Centre for Addiction Research in Childhood and Adolescence (DZSKJ), University Medical Centre Hamburg-Eppendorf, Martinistrasse 52, 20246 Hamburg, Germany; 2 Clinic for Psychiatry, Neurology, Psychosomatics and Psychotherapy in Children and Adolescents, University Medical Centre Rostock, Gehlsheimer Strasse 20, 18147 Rostock, Germany

## Abstract

**Background:**

Current research shows that overall numbers for cannabis use among adolescents and young adults dropped in recent years. However, this trend is much less pronounced in continuous cannabis use. With regard to the heightened risk for detrimental health- and development-related outcomes, adolescents and young adults with continuous cannabis use need special attention. The health services structure for adolescents and young adults with substance related problems in Germany, is multifaceted, because different communal, medical and judicial agencies are involved. This results in a rather decentralized organizational structure of the help system. This and further system-inherent characteristics make the threshold for young cannabis users rather high. Because of this, there is a need to establish evidence-based low-threshold help options for young cannabis users, which can be easily disseminated. Therefore, a training programme for young cannabis users (age 14-21) was developed in the "CAN Stop" project. Within the project, we seek to implement and evaluate the training programme within different institutions of the help system. The evaluation is sensitive to the different help systems and their specific prerequisites. Moreover, within this study, we also test the practicability of a training provision through laypersons.

**Methods/Design:**

The CAN Stop study is a four-armed randomized wait-list controlled trial. The four arms are needed for the different help system settings, in which the CAN Stop training programme is evaluated: (a) the drug addiction aid and youth welfare system, (b) the out-patient medical system, (c) the in-patient medical system and (d) prisons for juvenile offenders. Data are collected at three points, before and after the training or a treatment as usual, and six months after the end of either intervention.

**Discussion:**

The CAN Stop study is expected to provide an evidence-based programme for young cannabis users seeking to reduce or quit their cannabis use. Moreover, we seek to gain knowledge about the programme's utility within different settings of the German help system for young cannabis users and information about the settings' specific clientele. The study protocol is discussed with regard to potential difficulties within the different settings.

**Trial registration:**

ISRCTN: ISRCTN57036983

## Background

In 2008, the lifetime prevalence of cannabis use among German adolescents and young adults between age 12 and 25 of 28.3% dropped for the first time after a continuous rise throughout previous years (1997: 18.9%; 2001: 25.6%; 2004: 31.1%) [1]. This trend was confirmed by another large study throughout various European countries and North America in a survey of adolescents and their cannabis use over the past 12 months [2]. 12.8% of 15 year old boys and 10.2% of 15 year old girls reported having used cannabis at least once in the past 12 months in 2006, while rates in 2002 were higher for boys (22.3%) and girls (14.9%). These data, however, include occasional use of cannabis. Rates of a continuous use of cannabis, defined as more than ten occasions of cannabis use per year, also decreased in German 12 to 25 year-olds in 2008, yet the decrease was less pronounced and the prevalence of 2.3% [1] still shows, that continuous cannabis use remains a striking phenomenon among German adolescents and young adults.

Cannabis use is known to be associated with various psychosocial problems and negative outcomes for consumers. Best, Gross, Manning, Gossop, Witton and Strang [3] showed that adolescent cannabis users (age 14-16) spent more time with peers, who were involved in criminal activities and/or consumed cannabis and other substances (tobacco, alcohol and illicit drugs), while spending less time with parents. These phenomena tended to be more pronounced when adolescents initiated their cannabis use early within their life-span. Similar results were found by Flory, Lynam, Milich, Leukefeld and Clayton [4], who reported of a higher likelihood for getting arrested or showing symptoms of alcohol misuse or dependence and antisocial personality disorder among cannabis users. Once more, early-onset cannabis users were found to be at greater risk. In addition, marijuana users reported lower self-esteem, poorer family relations and peer pressure resistance than non-consuming controls. Poorer educational attainment was another negative outcome of adolescent and young adult marijuana users [5]. Adolescent drug and cannabis use was also linked to a less successful autonomy development and diminished perceived competences later in life [6]. Moreover, the strain for affected family members - especially parents - can be considerable when adolescent marijuana use becomes a habit in the family [7].

In addition, adolescent cannabis use was shown to be linked to psychiatric disorders. For once, marijuana use itself can result in cannabis misuse (ICD-10: F12.1) or dependence (ICD-10: F12.2). The existence of a physical withdrawal syndrome after marijuana detoxification is established among scientists [8]. Moreover, persons with a genetic risk for psychotic disorders tend to get diagnosed more often and earlier, after using marijuana. Other disorders, which are in discussion about being associated with cannabis use are depressive and anxiety disorders. Overall estimates of comorbid mental health problems are assumed to range as high as 70% in marijuana addicts [9]. Interestingly, high marijuana use patterns do not seem to be a necessary prerequisite for later mental health problems. Windle and Wiesner [5] already found elevated rates of anxiety disorders and major depression in young adults who reported "only" an experimental use of marijuana in their adolescence as compared to abstainers. In sum, mental health problems seem to be closely associated with both, experimental and continuous marijuana use. The timely sequence of marijuana use and other mental health problems is not fully understood yet, and therefore inferences about causal patterns can not be adequately substantiated. Evidence exists, that persons suffering from mental health problems may use cannabis as a means to regulate their emotions. On the other hand, marijuana use is known to be able to trigger e.g. panic attacks. Right now, multifactorial etiological models are used to explain the association between marijuana use and the various psychosocial and health-related outcomes. With regard to the multiple dysfunctional outcomes and health-risks, interventions are needed to reduce cannabis use and abuse in adolescents and young adults.

In Germany, the support system for drug- and addiction-related health problems consists of different institutions. The majority of institutions supporting individuals with drug-related problems belongs to the so-called addiction aid (Suchthilfe). Institutions of this kind are all supervised and funded by the communities (Kommunen). Mostly support is provided by independent welfare institutions, which receive their funding by the municipality and through private donations and which report to the local authorities. A broad network of these institutions exists mostly in the form of walk-in counselling facilities. Some in-patient projects exist, which provide e.g. accommodation and a pedagogic support system for long-term heavy drug users. The professional background among the staff varies ranging from social welfare workers, education specialists, psychologists, psychotherapists and nurses to physicians. As a result of this non-centralized set-up, a multitude of different approaches and concepts exists on how to deliver support for drug users. This enables communities to shape their support service alongside the specific needs of the municipality (e.g. with regard to their urban or rural backgrounds and related specific drug use patterns), yet, a lot of networking among different institutions is necessary to provide for an efficient exchange between institutions and adjacent authorities and organizations (e.g. legal authorities, police).

Individuals with drug-related problems may also approach the medical system for help. Costs for these services are covered by either private or public health insurances. All but 0.2% of Germans are covered by one of this kind of health insurances [10]. For out-patient support, a broad network of general practitioners and pediatrists exists, which either provide help themselves or refer patients to specialists such as psychiatrists, child and adolescent psychiatrists and psychotherapists. Patients can also choose to consult these specialists directly if they wish to. Some larger clinics also provide out-patient walk-in clinics for patients with mental health problems. Yet, only a few are specialized in treatment of adolescent and young adult drug abuse. For patients with a bigger need for help, day-clinics exist, with a psychiatric or child and adolescent psychiatric background. There too, mental health problems can be treated, however, again, specialized services to tackle substance abuse in concordance with other mental health problems are scarce and often, abstinence from drugs is a prerequisite for admission.

In-patient services for adolescent and young adult drug users are provided by psychiatric and child and adolescent psychiatric clinics. One of their main foci is to provide withdrawal treatment under medical supervision. Another task is to initiate a psychotherapeutic treatment after detoxification. Again, only few in-patient clinics exist with a specialized focus on drug using adolescents on own wards and concepts to combine withdrawal with subsequent psychotherapeutic treatment. Within the medical system, medical rehabilitation clinics stand out as a specialized agent of health care provision for patients with chronic diseases, among which drug abuse and addiction are also subsumed. Their main focus is to ensure the return of the chronically ill into the labour force. They are funded mainly through social pension insurance providers, less often by health insurances. Although adolescents may also be treated within the medical rehabilitation system for the chronically ill, specialized care regarding drug abuse or addiction exists only for individuals older than 17 years.

In sum, there are various institutions in Germany providing help for adolescents and young adults with marijuana abuse. Recently, the demand for help for this target group has been on the rise. Between 1999 and 2005, persons, who were seeking treatment for cannabis-related disorders, tripled in Europe [11]. The same trend was reported for Germany, where numbers of clients in out-patient counselling facilities, who named cannabis use as their main problem rose drastically [12] in the early 2000er years. As the number of continuous cannabis users still is rather high, this need is expected to continue. However, service and treatment provision for this special group turned out to be difficult for a number of reasons. A lot of walk-in counselling facilities, usually serving as the first contact point for persons seeking professional help, faced a sudden change during the early 2000es. With the rise of cannabis users, the age of help-seeking clients dropped. More than before, parents of affected teenagers sought help in walk-in counselling facilities. As a consequence, the profile of counselling facilities had to change with the new demand. Yet, experience was missing and help-seeking clients were partly deterred from existing counselling facilities. Clients did not feel they were in the right hands as traditional facilities carried the atmosphere of the traditional drug scene and its tendency to pauperization. To lower the threshold for possible new clients with cannabis-related problems, steps were initiated to link the facilities of the traditional addiction aid with facilities of the youth welfare context, a support system which for example run youth clubs, but previously identified not very much with the topic [13]. As an example, the City of Hamburg in 2006 opened five new walk-in counselling facilities with a special focus on adolescent substance use problems and started to link both addiction aid and youth welfare facilities in an innovative project. However, through the de-centralized set-up of the help system for young cannabis users in Germany, these kinds of initiatives remain up to local authorities and mostly do not base on guidelines developed by experts or on an evidence-based level. Still, the problem of getting access to young cannabis users with existing structures was identified by Federal agencies, which funded expertises on the problem [14].

With regard to the medical system, help-seeking adolescent and young adult cannabis users seemed to be rather reluctant to get counselling by physicians and psychotherapists. As described earlier, competent help could be given in the psychiatric and child and adolescent psychiatric field, however, this label seemed to deter both adolescents and their parents and young adults alike. Specialized out-patient clinics within the medical system, who manage to develop a tailored address to young cannabis users are rather limited and are difficult to reach for potential patients, especially in rural contexts. This is even more so, when motivation of patients is rather low. This, however, often is the case in cannabis users [14], who frequently show a limited awareness of their problem and a lack of drive. Consequently, the threshold to utilize services of the medical system is high and incoming patients are mostly those with protracted abuse histories.

In sum, with regard to the different institutions, which provide professional assistance for cannabis using adolescents and young adults, there are a multitude of offers. However, the facilities face a new challenge with the new group of help-seekers and have to respond to this demand rather quickly. Moreover, system-inherent conditions set the threshold for service utilization rather high.

Several efforts are made to rise to this challenge through several federally funded model-projects. Within these, standardized counselling and treatment programmes were developed or adapted and evaluated to provide for evidence-based offers. In the following, some of these projects will be introduced. The project CANDIS [15] refers to cannabis using patients 16 year old or older and encompasses ten psychotherapeutic single-client sessions with a cognitive behavioural therapeutic approach. First results are being published and evaluation was extended from a university-based psychological research centre to eleven walk-in counselling facilities. Treatment is delivered by specially trained psychologists.

The project INCANT adapted the multidimensional family therapy programme of Liddle and his research group [16] and combines family therapy with adolescent psychotherapeutic approaches. It is currently being evaluated in selected counselling facilities and comprises the whole family system of cannabis using clients aged 13 to 18.

Another programme, which was particularly developed for cannabis users (age 15 to 30), is named "Realize it!" and consists of several single- and group-counselling sessions. After a first evaluation in southern Germany and Switzerland, it is now extended to other walk-in counselling facilities in Germany [17]. FReD is a counselling programme, which is not tailored for cannabis users alone, but refers to persons aged 14 to 21, who got into legal conflicts with the police because of drug related offence and get invited to the programme as a consequence of their police record. Mirroring the nature of this concept, male participants of the programme are overrepresented. The programme encompasses eight group sessions and deals with drug-related problems as well as legal advice. An evaluation of the programme was undertaken [18] and the programme was finished its model phase in 2007 and is currently implemented at about 140 locations in Germany. "Quit the shit" is an internet-based counselling offer for adolescents and young adults with cannabis use problems. It comprises an online substance use diary and feedback from trained professionals (http://www.drugcom.de/bot_quittheshit.html) through chats. The approach to adolescent and young adult cannabis users turned out to be well-accepted. But researchers pointed out that it serves rather as a first step for cannabis users who seek help and may be inappropriate for aggravated consume patterns or cannabis users with additional mental health problems [19].

With the previously mentioned programmes on the agenda, however, several needs for the provision of comprehensive care for adolescent and young adult cannabis users remain unmet. So far, programmes with a specific focus on cannabis for adolescents and young adults are scarce, and existing programmes tend to rely on psychotherapeutically trained personnel or certain professional groups. Yet, these prerequisites for personnel are often not met by service providers, or they are not met in a sufficient number of existing staff members to face the rather high prevalence of help-seekers. With this regard, specific group-based offers seemed to be missing.

Another setting, in which systematic service provision beyond basic structural drug prevention efforts (e.g. general drug use ban etc.) for adolescent and young adult cannabis users is nearly entirely missing, is the juvenile penal system [20]. In Germany, juvenile prisons encompass persons aged 14 to 25 who were convicted under juvenile criminal law. Among the approximately 6500 mostly male young offenders, who are currently serving their prison sentence in Germany, nearly all have experiences with addictive drugs and 60% of incoming juvenile offenders report having used illicit drugs within the past four weeks before serving the prison sentence [21]. Among illicit drugs, cannabis is the most important problem drug [22] and although of course, cannabis use within juvenile prisons is strictly prohibited, ongoing consumption of cannabis takes place. Up to date, we know of no manualized or evidence-based support programme to address cannabis misuse or dependence in adolescent or young adult offenders serving their prison sentence.

In order to fill the described gaps in the support system for cannabis using adolescents and young adults, our research group developed a structured, manualized group training programme "CAN Stop", which focuses on this specific target group between age 14 and 21, whose needs so far have not been adequately met by the various support system components. The first goal of the project is to provide for an evaluated, evidence-based group programme, which can be easily disseminated and which is tailored to the developmental characteristics and contexts of adolescents and young adults. We hypothesize that young cannabis users who participated at the CAN Stop group training in addition to the treatment or service usually provided in the specific help provision context (e.g. drug addiction aid and youth welfare, in-patient medical context, out-patient medical context, juvenile prisons) show lower level of cannabis intake and higher levels of cannabis abstinence-related self-efficacy after the training as opposed to young cannabis users who received the usual treatment or service. As for the implementation of the programme, we are aware of the existing subdivided and at times confusing help provision system for young cannabis users in Germany, and we believe it is inherent for the effectiveness and for the future dissemination and practicability of the programme, that it is tried out within these different help provision settings. We hypothesize, that because of their above mentioned structural and organizational characteristics, different clientele and context-specific staff will be met within these settings. That is why the programme is implemented and evaluated separately in four different settings (a) the drug addiction aid and youth welfare system, (b) the out-patient medical system, (c) the in-patient medical system and (d) prisons for juvenile offenders. This offers the possibility to check for the suitability of the programme in different settings - our second goal - and to compare its effectiveness and utility within them. Our third goal is to draw conclusions for the future implementation of the CAN Stop training in different help provision settings. Moreover, our fourth goal is to gain knowledge about the incoming clientele of the different settings with regard to their substance use patterns, severity of dependence, psychosocial adjustment, expected effects of cannabis use and family and peer relations. As for the realization of the training programme, this remains in the hand of the existing staff of the different settings. A novelty of the programme is, that no specific prerequisites are prescribed as to the professional background or existing (therapeutic) experience of the trainers. The only exception is the staffs' contact with cannabis using adolescents and young adults as part of their occupation within one of the above mentioned settings and a basic interest to realize the training. This approach may include staff from prison guards, to ex-users working in the addiction aid system to psychotherapists and medical doctors and nurses. With this approach, the introduced study not only evaluates the effectiveness of a group training programme for cannabis using adolescents and young adults (14-21) but also evaluates the instructions given to the trainers, who perform the actual training with the target group, in form of a course of instruction and a newly developed manual. The fifth goal of the project is to evaluate the CAN stop group training programme with regard to its gender-specific utility and effectiveness. Previous therapeutic approaches were evaluated with largely male samples. This may in part be due to the higher prevalences of male cannabis users, but may to some extent also lie in the fact, that previous programmes and approaches were more shaped to address males and were not equally fit to address the specific needs of female cannabis users. The CAN Stop group training was explicitly developed to meet both male and female young cannabis users' needs in that it for example also refers to accompanying internalizing problems and the importance of the dynamics of a romantic partnership with cannabis use patterns - topics which are deemed to address especially female young cannabis users.

## Methods/Design

### Study Design and Setting

The CAN Stop study is a four-armed randomized wait-list controlled trial. Following Zwarenstein et al.'s [23] theoretical continuum of evaluation trials, on which rather "experimental", explanatory efficacy-oriented trials form the one end, and naturalistic, pragmatic effectiveness-oriented trials form the other end, this study tends towards the latter, pragmatic end. Developers were more interested in the real-life utility of the CAN Stop group training within the described four different contexts with their aforementioned specificities.

Data will be gathered at three points: before the group training (pre), after the group training (post; 3 months after pre) and at a follow-up (6 months after post). Modes of data retrieval vary from face-to-face interviews and questionnaires (pre), to questionnaires only (post) and telephone interviews and questionnaires (follow-up). Group comparisons will be made to check for the group training's effectiveness.

### Recruitment of cooperation partners and young cannabis users

Subjects for the study are recruited by cooperating institutions from the settings mentioned above. These cooperation partners were trained by the study group to identify incoming clientele for their eligibility to participate at the study, to perform motivating take-in talks to these young cannabis users, and to gather data for the first two points of measurement (pre and post). The follow-up measurement will be performed by the study group. Moreover, staff of the cooperation partners is instructed to perform the CAN Stop training.

In a first attempt to identify suitable cooperation partners, we systematically scanned comprehensive help provision and therapy guides of Northern and Eastern Germany as well as the Internet for institutions. All identified institutions were then invited to participate at the study with an information letter and asked to fax back an attached reply form together with a questionnaire about structural and work details of the institution, such as number of incoming young cannabis users in the targeted age range and number of staff. Prior to this, a target number of cooperation partners needed to gather the subjects was calculated on the basis of a power calculation, an estimated attrition rate of 50% and under the premise that CAN Stop training groups should be composed of six to ten group participants. If feedback was not given within six weeks after letters were sent out, a study group member called to check back with the institution. An exception from this procedure was made within the setting of juvenile prisons: because their total number was rather low, their staffs were contacted directly via phone calls. The response rate varied between different settings between 57% in juvenile prisons, 10% in in-patient medical clinics, 5% in out-patient medical clinics and 5% in the youth welfare and drug addiction aid. If institutions signalled to be in contact with young cannabis users in their daily routines and were interested in study participatioin, a study group member gave a presentation of the project and invited institutions to participate at the study. In sum, we could aquire a total of 11 cooperation partners within the youth welfare and drug addiction aid, 9 cooperation partners of the out-patient medical system, 8 cooperation partners of the in-patient medical system and 3 participating prisons for juvenile offenders. Participating cooperation partners were randomly assigned to perform the CAN Stop intervention plus their usual treatment as an intervention group or to provide for control group subjects, who received the cooperation partner's previously installed treatment as usual (TAU). As for other treatments, provided by the different institutions (besides CAN Stop), cooperation partners differed in their usually provided routines depending on their specific setting. While most prisons indicated, other kinds of therapy besides CAN Stop were rather limited and merely consisted of counselling talks or conceptual work within the organizational framework of the prison, the in-patient medical system's cooperation partners of course provided many other facets of treatment - to mention both extremes of this varying continuum. Institutions, which indicated they were already participating in other structured programmes similar to CAN Stop were excluded from the study in order not to compare CAN Stop with eventually competing studies (e.g. FrED, see above).

Recruitment for subjects was performed by cooperation partners within their daily work routines. In addition, the study group provided for flyers, which gave information on the study, its objectives and the group training to recruit participants. Young cannabis users interested in the programme were asked to contact a centralized telephone hotline installed by the study group to provide for a first check-up of inclusion criteria, to inform about the study and then to randomly allocate subjects to wait list control groups or to intervention groups in respective institutions. Young cannabis users in prisons are exempted from this procedure, because a random allocation of prisoners is not possible: the juidical allocation of young offenders into the prisons will decide about whether they are selected into a prison performing an intervention or a control group, their inclusion criteria are checked in in-take talks. Inclusion criteria were (1) the participants' age between 14 and 21 years, (2) a cannabis consume, which is deemed problematic by the young cannabis user or significant persons in his or her context (e.g. parents, teachers) (3) the participant's willingness to at least think over previous consumption patterns and to participate at a group dealing with the topic and (4) the informed consent of participants and in the case of underage participants the informed consent of a parent or official guardian. Young cannabis users were excluded from the study when they showed acute symptoms of psychosis or suicidality. Both of these exclusion criteria were checked via standardized screening questions at the beginning of the face-to-face take-in talks at the respective cooperation partner's institutions. Young cannabis users were invited to participate at the study regardless of whether their access to cooperating institutions was initiated by the cannabis users themselves or whether pressure was exerted by e.g. school, employers or because of a judicial condition of probation, however, this is documented and will be integrated into outcome analyses.

Both, staff from the cooperating institutions and study participants received incentives for their efforts or taking part in the study, respectively. Data gathering for pre and post measurement by a staff member of the cooperation partner is awarded 10€, the performance of a complete training cycle is compensated with 400€. Young cannabis users receive 10€ for the completion of the pre measurement, 25€ for the completion of the post measurement and 50€ for the completion of a follow-up measurement. Ethical questions arose, whether it is suitable to give out cash to young cannabis users, given the possibility that the money is spent for cannabis. However, we deemed the sum of the incentives for young cannabis users small enough to be compared with money, adolescents and young adults handle in their daily routines and therefore argued that no additional allurement is attached to the incentives. Moreover, previous research with our institution had shown, that cash incentives were more potent and appealing for study subjects (Ravens-Sieberer, personal communication). If cooperation partners severely objected to this procedure, however, we left the ultimate decision about the nature of incentives to their staff and instructed them to give out gift vouchers e.g. for local music stores instead.

### Intervention: The CAN Stop group training

The CAN Stop group training consists of eight group sessions à 90 minutes. Groups consist of six to ten young cannabis users and one or two trainers. The group contents and its timeline is described in a detailed manual. Moreover, a general introductory section of the manual provides trainers with background knowledge about cannabis, its effects, aspects of group dynamic as well as recommendations regarding the trainer's attitude towards participants and their consumption. The training's rationale is based on behavioural therapeutic and motivational interviewing elements [24]. It encompasses consumer or craving diaries, the work with social and emotional context variables and potential triggers as well as the elaboration of alternative behaviour strategies and the activation of participants' resources. Generally and for motivational reasons, it is left open to participants within the training whether they want to reduce or stop their cannabis use. However, certain settings at which the training is tested officially require absolute consume stops (e.g. prisons), yet this is a characteristic of the respective institution and not of the training itself. A more detailed description of the training is in preparation. After completing the CAN Stop training, certificates are handed out to participants who, participated at least at five out of eight group training sessions, for further gratification. If within a group, only three or less participants remain after other participants dropped out of the group, the group is not further continued.

### Training of CAN Stop trainers

The CAN Stop training is performed by cooperation partners' staff. Their professional background may vary starkly with regard to their therapeutical expertise. In order to empower cooperation partners' staff for performing the training, motivating take-in talks and procedures for data gathering, a detailed training is set up, which every trainer has to undergo beforehand. As an effect, our study's goal in essence is, not only to test the effect of the CAN Stop training but also to test the approach to empower cooperations partners' staff to perform the training. Several measures are taken to provide for a sufficient quality assurance with regard to the provision of the CAN Stop training. CAN Stop trainers are asked to record each session on videotape. Videotapes will later be used to check for trainers' adherence to the manual. A special adherence measure was developed, which is oriented closely to the training contents of each session. Regular supervision phone calls with trainers are made by the study group to ensure trainers feel comfortable with their CAN Stop training delivery, and trainers are invited to phone the study group if questions arise, be it regarding contents or regarding difficult social situations within the training or when training participants show signs of acute psychosis or suicidality. Notes are taken of these supervision talks for later qualitative analyses. Identification of these problems and recommendations about how to deal with them are also part of the training of trainers. For further control with regard to the young cannabis users, participants' comfort with the training, participants are asked to indicate their mood shortly at the beginning and end of each training sessions with colour-coded cards. Moreover, participants give feedback on the respective session at the end of each CAN Stop training session with regard to their satisfaction with the training contents and the atmosphere in the group. These data will be gathered and monitored by the study group to ensure that no CAN Stop training runs out of hand.

### Measures

Young cannabis users participating at the study were asked to fill in several measures to assess target variables. Table 1 gives an overview of measures used.

**Table 1:** Measures for young cannabis users

Target variable	Measure	Point of measurement
Sociodemographics Living situation Social network Problems with school, police, debts... Education Prior counselling and therapies		pre, follow-up
Screening for acute psychosis	Subscale of the Diagnostisches Interview psychischer Störungen (DIPS; Diagnostic Interview of psychiatric disorders) [26]	pre
Substance use history	Following the assessment standards III of the DG-Sucht (German Society for Addiction Research and Therapy)	pre, post, follow-up
Severity of dependence	Severity of dependence scale (SDS) [27]	pre, post, follow-up
Psychosocial adjustment	Youth self report (participants aged 14-17) [28] Young adult self report (Participants aged 18-21) [29]	pre, post, follow-up
Expected positive and negative effects of cannabis use	Comprehensive Cannabis Expectancy Questionnaire (CCEQ) [30]	pre, post, follow-up
Relationship to friends and peers	Questionnaire for health-related quality of life (Kiddo-KINDL revised, subscale "friends") [31]	pre, post, follow-up
Family relationships	Familienbogen/Selbstbeurteilungsbogen (family inventory, self-rating) [32]	pre
Motivation for change in cannabis use	Fragebogen zur Erfassung der Veränderungsbereitschaft (FEVER; Questionnaire to protocol the willingness for change) [33,34]	pre, post, follow-up
Personal goals with regard to changes in substance use	Zielskala [35]	pre, post, follow-up
Self-efficacy with regard to cannabis abstinence or limited cannabis use	Heidelberger Skalen zur Abstinenz-/Kontrollzuversicht (HEISA-16/HEIS-KOTZ-12; Heidelberg scales of confidence regarding abstinence/controlled use) [35]	pre, post, follow-up
Satisfaction with the training	Fragebogen zur Beurteilung der Behandlung, Version Patient (FBB-P; Questionnaire for the satisfaction with treatment, patient version) with minor adaptations [36]	post
Peer resistance regarding cannabis use	own development	pre, post, follow-up

In addition, trainers were asked to answer a short questionnaire about themselves prior to t0 in order to gather data for analysing possible trainer effects in secondary analyses. This included their professional background, own beliefs and attitudes towards cannabis use and personality characteristics, using the Big Five Inventory-10 (BFI-10) [25].

### Measures against bias

The study is unblinded with regard to trainers, who hand out the questionnaires at t0 and t1 measurements to the clients. However, t2 telephone interviewers are blind to whether participants belonged to a control or intervention group. A problem of the study may be different selection effects in the groups. Participants of the study may be inclined to follow through a regular training as opposed to potentially less structured treatment as usual. In order to estimate this kind of bias we will analyze models to predict drop-outs in both, the intervention and the control group. In order to prevent trainers from using CAN Stop training elements in their working routine and to unintentionally blur TAU and CAN Stop interventions, trainers of institutions, who were selected into the control group will receive their training for the CAN Stop training intervention only after they completed t1 measurements in young cannabis users. Supervision protocols of CAN Stop trainers for later qualitative analyses will be handled in a way that no inference can be made about the respective trainer.

### Ethical considerations

All procedures are in concordance with the ethics committee of the Chamber of Physicians in all federal states of Germany, in which the CAN Stop study takes place. All participants of the study and - in the case of underage young cannabis users - their parents or official guardians are informed about the study goals, procedures, analyses and data reporting prior to participation. Only data of participants who gave written consent will be used for analyses. All young cannabis users of the control group are offered the CAN Stop training after their data gathering is completed. In order to protect young cannabis users' identity to the study group personnel, videographies of CAN Stop training sessions will be made in a way that the camera is focused solely on the CAN Stop trainer - so only voices of young cannabis users are audible on videotapes.

### Analysis

An intention-to-treat approach will be taken to analyze data. Comparisons will be made between intervention group and the TAU control group in the different settings using baseline controlled ANCOVAs with cannabis use and abstinence-oriented self-efficacy as dependent variable and cannabis use at t0 as covariate. We plan to perform nested analyses in order to handle the variance, which is shared between participants attending the same institution. There are no institutions, which offer more than one CAN Stop training group, so a further layer of nesting is not nescessary. We hypothesize that intervention groups show lower levels of cannabis use and higher levels of self-efficacy related to cannabis-abstinence after the intervention as compared to controls. To discover these effects, we calculated the needed sample size of n = 119, assuming a medium effect size of f = 0.25, given the power of the statistical test to .80 and setting the α-error on .05. Prior experience with the target group have shown that attrition rates among young cannabis users are rather high, so we calculated with an attrition rate of 50% when planning the number of nescessary participants at t0. With regard to further effects, we believe there are differences in the effectiveness of the training in the different settings. We hypothesize that the effect of the training (d) will be greater when less other interventions take place in the respective setting, that is, effects are bigger in juvenile prisons and out-patient institutions than in in-patient setting clinics.

Secondary analyses will be made to compare the clientele of the different settings. Here we assume that more aggravated and chronic cannabis users will be found in prisons and in-patient settings than in out-patient settings. As for the role forced access to the group (e.g. because of pressure from juidical institutions or teachers) plays, we believe no differences will be seen between participants, who indicated, they were attending the intervention group because of pressure from outside, as compared to participants, who decided to attend the group solely on their own accord. This is, because we assume the CAN Stop training itself is shaped in a way as to leave decisions about further consume open to participants, which inspires the internal motivation of all participants and therefore blurs differences regarding forced access to the training.

In order to gain insight into the training's utility in the different settings, qualitative and quantitative feedback data from trainers will be synthesized and discussed with clinical experts from different settings for triangulation. Conclusions from this discussion process will be shown to trainers in order to test the plausibility of our conclusions to trainers and to check whether they feel their experiences are sufficiently mirrored in the conclusions.

## Discussion

In this paper, we describe the study design of an innovative training targeting young cannabis users in rather different settings, which are very differently organised but at the same time all share the goal to reduce their clientele's prior cannabis use. We are especially interested in how the different institutions will be able to implement the training, what the differences between their respective study participants are and how this is mirrored in the effect(s) of the training. A special difficulty of the study was to set up a study procedure which matches the different frameworks of the respective setting, starting at the professional background of the cooperation partners' staff, their legal and organizational background and their different interests.

Several difficulties may arise when it comes to the implementation of the study plan. First, it remains to be seen, whether it is suitable to use the centralized study telephone as a means to randomize young cannabis users into different institutions, especially, when they have already had contact to workers in one institution e.g. through a personal talk when handing out the study flyer, and they are assigned to another institution because the respective group is available only there. It remains to be seen, whether this potential switch marks a problem for recruitment, because other institutions are less easy to reach (e.g. with public transportation in rural areas) or an attachment has already been formed between one institution and a young cannabis user.

Another problem may arise through the referral of young cannabis users to other institutions by the centralized study hotline, because an institution, which may already have established a first contact to a young cannabis user, could "loose" a potential client to another, possibly competing institution and therefore may feel to loose money usually granted to them when treating the client. We hope that we can convince all cooperation partners that statistically, the possibility to "loose" clients through the centralized telephone hotline is as high as is the possibility to gain new clients because of the same centralized referral of study participants.

If it turns out, that the procedure with regard to the centralized telephone hotline poses a major threat to the recruitment of study participants, we will discuss to switch into a cluster randomized study design with cooperating institutions as randomized subjects.

What may be another weakness of the study is the different dynamic of group - in our case the CAN Stop training group - as opposed to individual treatment in the case of individual TAUs. Groups may be terminated if too many participants quit the group and therefore, even participants, motivated to follow through the CAN Stop training may be faced with a forced dismissal of the intervention. This effect is not prevalent in individual trainings and result in different attrition effects between the intervention and the control group. Moreover, unspecific group effects of the training may not be sufficiently controlled through this study design.

We take these weaknesses into account, however, because we wanted to remain rather close to the everyday working routine of our cooperation partners and a rather pragmatic approach is nescessary to tackle the problem of young cannabis users in a timely and economic fashion.

We believe that it is nescessary to test specific intervention effects in real-life as opposed to rather artificial but, potentially methodologically more pure approaches. Of course, we know about this characteristic of the CAN Stop study and will take care of the mentioned problems when analysing our data and discussing the results.

## Competing interests

The authors declare that they have no competing interests.

## Authors' contributions

CB designed the study and its methodology, coordinates the study and drafted the manuscript of this paper. AM, NW and KM participated in designing the study and carry through its organizational processes and cooperations. OR and RT conceived of the study, and participated in its design and coordination. All authors read and approved the final manuscript.

## Pre-publication history

The pre-publication history for this paper can be accessed here:

http://www.biomedcentral.com/1472-6963/11/80/prepub

## References

[B1] Bundeszentrale für gesundheitliche Aufklärung (BZgA) Die Drogenaffinität Jugendlicher in der Bundesrepublik Deutschland 2008 Köln: BZgA

[B2] Kuntsche E Simons-Morton B Fotiou A ter Bogt T Kokkevi A Decrease in Adolescent Cannabis Use From 2002 to 2006 and Links to Evenings Out With Friends in 31 European and North American Countries and Regions Arch Pediatr Adolesc Med 2009 163 2 119 125 10.1001/archpediatrics.2008.542 19188643 PMC2646163

[B3] Best D Gross S Manning V Gossop M Witton J Strang J Cannabis use in adolescents: the impact of risk and protective factors and social functioning Drug Alcohol Rev 2005 24 6 483 488 10.1080/09595230500292920 16361204

[B4] Flory K Lynam D Milich R Leukefeld C Clayton R Early adolescent through young adult alcohol and marijuana use trajectories: Early predictors, young adult outcomes, and predictive utility Dev Psychopathol 2004 16 1 193 213 10.1017/S0954579404044475 15115071

[B5] Windle M Wiesner M Trajectories of marijuana use from adolescence to young adulthood: Predictors and outcomes Dev Psychopathol 2004 16 4 1007 1027 10.1017/S0954579404040118 15704825

[B6] Chassin L Pitts SC DeLucia C The relation of adolescent substance use to young adult autonomy, positive activity involvement, and perceived competence Dev Psychopathol 1999 11 4 915 932 10.1017/S0954579499002382 10624732

[B7] Küstner U Baldus C Motivationsbehandlung eines Adoleszenten mit einer substanzbedingten Störung Psychotherapeut 2009 54 205 210

[B8] Budney AJ Vandrey RG Hughes JR Thostenson JD Bursac Z Comparison of cannabis and tobacco withdrawal: Severity and contribution to relapse J Subst Abus Treat 2008 35 4 362 368 10.1016/j.jsat.2008.01.002 PMC4345250 18342479

[B9] Thomasius R Weymann N Stolle M Petersen KU Cannabiskonsum und -missbrauch bei Jugendlichen und jungen Erwachsenen Psychotherapeut 2009 54 170 178 10.1007/s00278-009-0662-x

[B10] Statistisches Bundesamt Fachserie 13 Reihe 1.1 - Sozialleistungen, Angaben zur Krankenversicherung (Ergebnisse des Mikrozensus) 2008 Wiesbaden: Statistisches Bundesamt

[B11] European Monotoring Centre for Drug and Drug Addiction (EMCDDA) A cannabis reader: global issues and local experiences. Perspectives on cannabis controversies treatment and regulation in Europe 2008 Lissabon: EMCDDA

[B12] Simon R Sonntag D Cannabisbezogene Störungen: Umfang, Behandlungsbedarf und Behandlungsangebot in Deutschland 2004 München: IFT-Institut für Therapieforschung

[B13] Bange D Kristian S Thiem M Thomasius R, Schulte-Markwort M, Küstner UJ, Riedesser P Jugend- und Familienhilfe Suchtstörungen im Kindes- und Jugendalter: Das Handbuch - Grundlagen und Praxis 2008 Stuttgart: Schattauer 413 417

[B14] Görgen W Hartmann R Expertise Zugang zu jungen CannabiskonsumentInnen 2006 Münster: Landschaftsverband Westfalen-Lippe & FOGS

[B15] Hoch E Zimmermann P Noack R Rohrbacher H Pixa A Henker J Dittmer K Bühringer G Wittchen HU Thomasius R, Petersen KU Manualisierte einzeltherapeutische Behandlung problematischen Cannabiskonsums: Die "CANDIS"- Studie Cannabismissbrauch und -abhängigkeit 2009 Lengerich: Pabst

[B16] Liddle HA Dakof GA Parker K Diamond GS Barret K Tejada M Multidimensional Family Therapy for adolescent substance abuse: Results of a randomized clinical trail Americam Journal of Drug & Alcohol Abuse 2001 27 651 687 10.1081/ada-100107661 11727882

[B17] Hubrich R Hüsler G Minder W Tossmann P Realize it! Beratung bei Cannabiskonsum SuchtMagazin 2008 3 34 38

[B18] Görgen W Hartmann R Olivia H Frühintervention bei erstauffälligen Drogenkonsumenten (FreD) - Ergebnisse der wissenschaftlichen Begleitung 2003 Köln: Gesellschaft für Forschung und Beratung im Gesundheits- und Sozialbereich

[B19] Tossmann P Forschung und Praxis der Gesundheitsförderung, Band 31. Neue Wege in der Prävention des Drogenkonsums - Onlineberatung am Beispiel von drugcome.de 2007 Köln: BZgA

[B20] Thiel A Thomasius R, Schulte-Markwort M, Küstner UJ, Riedesser P Suchtprävention im Jugendstrafvollzug Suchtstörungen im Kindes- und Jugendalter: Das Handbuch - Grundlagen und Praxis 2008 Stuttgart: Schattauer 406 409

[B21] Kriminologischer Dienst im Bildungsinstitut des niedersächsischen Justizvollzugs Drogenerfahrungen von Inhaftierten im niedersächsischen Justizvollzug - Ergebnisse einer Zugangs- und Stichtagserhebung 2006 Celle: Kriminologischer Dienst

[B22] Köhler D Psychische Störungen bei jungen Straftätern 2004 Hamburg: Kovac

[B23] Zwarenstein M Treweek S Gagnier JJ Altman DG Tunis S Haynes B Oxman AD Moher D Improving the reporting of pragmatic trials: an extension of the CONSORT statement Br Med J 2008 337 12 10.1136/bmj.a2390 PMC3266844 19001484

[B24] Miller WR Rollnick S Motivational Interviewing - preparing people for change 2002 New York: The Guilford Press

[B25] Rammstedt B John OP Measuring personality in one minute or less: A 10-item short version of the Big Five Inventory in English and German J Res Pers 2007 41 1 203 212 10.1016/j.jrp.2006.02.001

[B26] Schneider S Marggraf J Diagnostisches Interview psychischer Störungen - 3. vollständig überarbeitete Auflage 2005 Heidelberg: Springer

[B27] Gossop M Darke S Griffiths P Hando J Powis B Hall W Strang J The Severity of Dependence Scale (Sds) - Psychometric Properties of the Sds in English and Austrian Samples of Heroin, Cocaine and Amphetamine Users Addiction 1995 90 5 607 614 10.1111/j.1360-0443.1995.tb02199.x 7795497

[B28] Arbeitsgruppe Deutsche Child Behaviour Checklist Fragebogen für jugendliche; deutsche Bearbeitung des Youth Self-Reports (YSR) der Child Behaviour Checklist. Einführung und Anleitung zur Handauswertung. 2. Aufl. mit deutschen Normen, bearbeitet von Döpfner M, Plück, J, Bölte, S, Lenz, K, Melchers P, Heim, K 1998 Köln: Arbeitsgruppe Kinder-, Jugend- und Familiendiagnostik (KJFD)

[B29] Achenbach TM Manual for the Young Adult Self-Report and Young Adult Behavior Checklist 1997 Burlington: University of Vermont Department of Psychiatry

[B30] Jäschke J Cannabiswirkungserwartungen: Entwicklung des Comprehensive Cannabis Expectancy Questionnaire (CCEQ) 2006 Münster: Westfälische Wilhelms-Universität

[B31] Ravens-Sieberer U The revised KINDL-R. Final results on reliability, validity and responsiveness of a modular HRQOL instrument for children and adolescents Quality of Life Research 2001 10 199

[B32] Cierpka M Frevert G Die Familienbögen. Ein Inventar zur Einschätzung von Familienfunktionen. Handanweisung 1994 Göttingen: Hogrefe

[B33] Hasler G Klaghofer R Buddeberg C The University of Rhode Island Change Assessment Scale (URICA) psychometric testing of a German version Psychother Psychosom Med Psychol 2003 53 9-10 406 411 14528410 10.1055/s-2003-42172

[B34] Hasler G R K Buddeberg C Der Fragebogen zur Erfassung der Veränderungsbereitschaft (FEVER) - Testung der deutschen Version der University of Rhode Island Change Assessment Scale (URICA) Psychother Psychosom Med Psychol 2001 53 406 411 10.1055/s-2003-42172 14528410

[B35] Körkel J Schindler C Hannig J Glöcknier-Rist A, Rist F, Küfner H Die Heidelberger Skalen zur Abstinenzzuversicht (HEISA) Elektronisches Handbuch zu Erhebungsinstrumenten im Suchtbereich (EHES). Volume 3.00 2003 Mannheim: Zentrum für Umfragen, Methoden und Analysen

[B36] Mattejat F Remschmidt H Fragebogen zur Beurteilung der Behandlung (FBB). Manual 1999 Göttingen: Hogrefe

